# Comparing angiotensin receptor–neprilysin inhibitors with sodium–glucose cotransporter 2 inhibitors for heart failure with diabetes mellitus

**DOI:** 10.1186/s13098-023-01081-2

**Published:** 2023-05-26

**Authors:** Ming-Lung Tsai, Yuan Lin, Ming-Shyan Lin, Tzu-Hsien Tsai, Ning-I Yang, Chao-Yung Wang, I-Chang Hsieh, Ming-Jui Hung, Tien-Hsing Chen

**Affiliations:** 1 Division of Cardiology, Department of Internal Medicine, New Taipei Municipal TuCheng Hospital, New Taipei City, Taiwan; 2 Chang Gung University College of Medicine, Taoyuan, Taiwan; 3 Department of Emergency Medicine, Keelung Chang Gung Memorial Hospital, Keelung, Taiwan; 4 Division of Cardiology, Department of Internal Medicine, Chiayi Chang Gung Memorial Hospital, Chiayi, Taiwan; 5 Division of Cardiology and Department of Internal Medicine, Ditmanson Medical Foundation Chiayi Christian Hospital, Chiayi, Taiwan; 6 Division of Cardiology, Department of Internal Medicine, Keelung Chang Gung Memorial Hospital, Keelung, Taiwan; 7 Division of Cardiology, Department of Internal Medicine, Linkou Chang Gung Memorial Hospital, Taoyuan, Taiwan

**Keywords:** ARNI, SGLT2i, Heart failure, Renal function, Diabetes mellitus

## Abstract

**Background and aims:**

Clinical comparisons of angiotensin receptor–neprilysin inhibitors (ARNI) and sodium–glucose cotransporter 2 inhibitors (SGLT2i) treatment in patients with HFrEF and T2DM are limited. This study evaluated the clinical outcomes and treatment benefits of SGLT2i versus ARNI treatment in patients with HFrEF and T2DM in a large real-world data set.

**Methods:**

We identified 1487 patients with HFrEF and T2DM who were undergoing ARNI or SGLT2i treatment for the first time (*n* = 647 and 840, respectively) between January 1, 2016, and December 31, 2021, and with clinical outcomes of CV death, hospitalization for heart failure (HHF), composite CV outcomes, or renal outcomes.

**Results:**

The HHF risk reduction conferred by SGLT2i treatment was more significant than that conferred by ARNI treatment (37.7% vs. 30.4%; 95% confidence interval [CI] 1.06–1.41). SGLT2i use conferred significantly greater renal protection against the doubling of serum creatinine (13.1% vs. 9.3%; 95% CI 1.05–1.75), an estimated glomerular filtration rate decline of > 50% (24.9% vs. 20.0%; 95% CI 1.02–1.45), and progression to end-stage renal disease (3.1% vs. 1.5%; 95% CI 1.62–5.23). The improvements in echocardiographic parameters were comparable between the groups.

**Conclusions:**

Compared with ARNI treatment, SGLT2i treatment was associated with a more significant HHF risk reduction and greater preservation of renal function in patients with HFrEF and T2DM. This study also supports the prioritization of SGLT2i use in these patients when patients' conditions or economic resources need to be considered.

**Supplementary Information:**

The online version contains supplementary material available at 10.1186/s13098-023-01081-2.

## Introduction

Concurrent heart failure with reduced ejection fraction (HFrEF) and type II diabetes mellitus (T2DM) is associated with higher cardiovascular (CV) mortality risks and hospitalization for heart failure (HHF) [1]. Angiotensin-converting enzyme inhibitors (ACEI) are fundamental treatments for HFrEF [2, 3]. Angiotensin receptor–neprilysin inhibitors (ARNI) are a new treatment standard established on the foundation of ACEI. The Prospective Comparison of ARNI with ACEI to Determine the Impact on Global Mortality and Morbidity in Heart Failure Trial (PARADIGM-HF) enrolled patients with chronic heart failure (mean age: 63.8 years) and an average left ventricular ejection fraction of 29.6%. A significant composite CV risk reduction of 20% with ARNI compared with enalapril treatment (hazard ratio [HR] 0.80; 95% confidence interval [CI] 0.73–0.87; *P* < 0.001) was reported, as was a significantly lower HHF rate (HR 0.79; 95% CI 0.71–0.89; *P* < 0.001) [4].

Sodium–glucose cotransporter 2 inhibitors (SGLT2i), a novel treatment for HFrEF, were initially taken in medication for T2DM. Their clinical application was expanded after their CV benefits were demonstrated in several trials [5–7]. The DAPA-HF trial, which enrolled patients with HFrEF with or without T2DM, reported that treatment with dapagliflozin was associated with a 30% risk reduction of HHF (HR 0.70; 95% CI 0.59–0.83) and a 26% (HR 0.74; 95% CI 0.65–0.85; *P* < 0.001) risk reduction of composite CV outcomes [8]. The EMPEROR-Reduced trial demonstrated a 30% risk reduction of HHF (HR 0.70; 95% CI 0.58–0.85; *P* < 0.001) and a 25% risk reduction of composite CV outcomes (HR 0.75; 95% CI 0.65–0.86; *P* < 0.001) [9].

ARNI and SGLT2i were both listed as Class I indications for HFrEF in the 2021 European Society of Cardiology and 2022 American College of Cardiology and American Heart Association Joint Committee heart failure guidelines [10, 11]. SGLT2i treatment was suggested for patients with both HFrEF and T2DM. However, Clinical evidence related to and comparisons of ARNI and SGLT2i treatments in patients with HFrEF and T2DM are limited. Moreover, because of the lack of evidence and high medication costs, simultaneously initiating both types of treatment is often unavailable. Therefore, using a large, real-world data set, we evaluated the clinical outcomes and benefits of SGLT2i versus ARNI treatment in patients with concurrent HFrEF and T2DM.

## Methods

### Data source

This retrospective cohort study was conducted using the Chang Gung Research Database (CGRD), a deidentified database managed by the Chang Gung Memorial Hospital (CGMH) healthcare system, the largest healthcare provider in Taiwan. The CGMH system is multi-institutional, comprising seven healthcare institutions (including four tertiary academic medical centers) across Taiwan. The Institutional Review Board of CGMH approved the study protocol and waived the requirement for informed consent. The patients’ records were anonymized and deidentified before analysis. Details regarding the CGRD have been published elsewhere [12, 13]. This study was conducted in accordance with the tenets of the Declaration of Helsinki [14].

### Study population and cohort

**Fig. 1:**
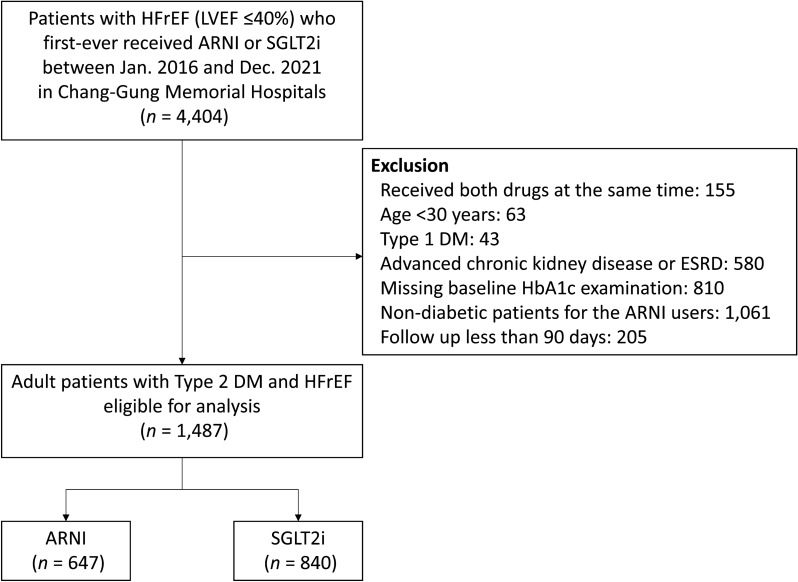
Study flowchart. *ARNI* angiotensin receptor–neprilysin inhibitor, *DM* diabetes mellitus, *ESRD* end-stage renal disease, *HbA1c* glycohemoglobin, *HFrEF* heart failure with reduced ejection fraction, *SGLT2i* sodium–glucose cotransporter 2 inhibitors

We retrieved the records of T2DM patients diagnosed with heart failure with a left ventricular ejection fraction (LVEF) ≤ 40% who received ARNI or SGLT2i treatment for the first time between January 1, 2016, and December 31, 2021. The index date was the date on which each patient received ARNI or SGLT2i after their HFrEF was diagnosed; therefore, we adopted a new-user design. The LVEF of each patient 6 months prior to the index date was determined through M-mode echocardiography or Simpson’s method. Patients were excluded if they were aged younger than 30 years; had a diagnosis of type I DM; had advanced chronic kidney disease (CKD), as indicated by an estimated glomerular filtration rate (eGFR) of < 30 mL/min/1.73 m^2^; had end-stage renal disease requiring chronic dialysis; or had missing baseline glycohemoglobin (HbA1c) data. In addition, patients without diabetes, as defined by a baseline HbA1c of < 6.5% and the non-use of anti-diabetic drugs were excluded. Patients whose follow-up periods were < 90 days were also excluded (Fig. 1) Additional file 1: Fig. S1A and Additional file 2: Fig. S1B.

### Covariate measurements and outcome definitions

The covariates of interest were demographic characteristics, baseline vital signs, HHFs history, comorbidities, medications used during the treatment period, laboratory test results, and echocardiography results. Clinical events comprised CV and renal outcomes. CV outcomes were a composite of CV death or HHF, HHF, CV death, all-cause mortality, myocardial infarction, and ischemic stroke. Renal outcomes comprised doubling of serum creatinine, an eGFR decline of > 50%, end-stage renal disease (ESRD), and hyperkalemia (potassium of > 6 mEq/L) during follow-up. A detailed definition of other covariate used in this analysis and outcome definition is listed in the Additional file 3.

### Statistical analysis

A substantial between-group difference in baseline demographics and clinical characteristics was observed. Therefore, inverse probability treatment weighting (IPTW) based on a propensity score was conducted to balance the baseline data between the two groups. The propensity score was estimated using the generalized boosted model (GBM) on the basis of 100,000 regression trees [15]. The baseline data presented in Table 1 were included in the propensity score calculation. The balance between groups before and after GBM-IPTW was assessed using standardized differences (STDs), which is not relevant to the sample size. An absolute value of < 0.2 indicated a non-substantial difference between groups. Single imputation using an expectation–maximization algorithm was employed to account for the substantial number of missing values in the continuous baseline data. All outcome comparisons were made in the complete imputed data and IPTW-adjusted cohort. Analyses were performed using SAS software, Version 9.4 of the SAS System for Unix (SAS Institute, Cary, NC, USA). A two-sided *P* value of < 0.05 was considered significant. A further detailed statistical analysis was described in the Additional file 3.

**Table 1:** Baseline demographics and clinical characteristics of patients before inverse probability treatment weighting

Variable	Available	Before GBM-IPTW^a^			After GBM-IPTW^b^		
	Number	ARNI	SGLT2i	STD^c^	ARNI	SGLT2i	STD^c^
Number of patients	1.487	647	840	–	1192.6	1284.5	–
Age, year	1.487	64.3 ± 13.3	63.0 ± 12.4	0.10	63.9 ± 12.9	63.5 ± 12.5	0.04
Male	1.487	504 (77.9)	646 (76.9)	0.02	76.4	76.8	− 0.01
Smoke	1.487	237 (36.6)	306 (36.4)	< 0.01	36.9	35.3	0.03
Body mass index, kg/m^2^	1.196	26.6 ± 4.9	26.2 ± 4.7	0.08	26.6 ± 5.0	26.2 ± 4.7	0.09
Baseline vital sign							
Systolic blood pressure, mmHg	1.346	126.9 ± 21.9	127.1 ± 22.8	− 0.01	128.3 ± 22.6	126.7 ± 23.0	0.07
Diastolic blood pressure, mmHg	1.346	73.4 ± 14.0	73.8 ± 14.0	− 0.03	73.7 ± 13.9	73.5 ± 14.0	0.02
Heart rate, bpm	1.345	81.2 ± 16.2	83.6 ± 16.0	− 0.15	81.6 ± 16.1	83.3 ± 16.2	− 0.10
Echocardiography result							
LVEF, %	1.487	29.0 ± 6.5	31.6 ± 6.8	− 0.38	29.8 ± 6.6	31.0 ± 6.8	− 0.17
LVEDD, mm	1.487	61.7 ± 8.5	58.2 ± 8.0	0.43	60.6 ± 8.4	59.1 ± 8.0	0.19
LVESD, mm	1.485	52.6 ± 9.4	48.4 ± 8.2	0.48	51.4 ± 9.6	49.3 ± 8.3	0.23
LA diameter, mm	1.486	45.2 ± 7.7	43.5 ± 8.0	0.23	44.9 ± 7.6	43.7 ± 8.0	0.15
MR severity	1.487						
Severe		31 (4.9)	34 (4.1)	0.04	4.54	4.47	< 0.01
Moderate		156 (24.5)	134 (16.0)	0.21	23.2	17.2	0.15
Mild		367 (57.5)	527 (63.0)	− 0.11	58.5	62.1	− 0.08
Trivial/None		84 (13.2)	141 (16.9)	− 0.10	13.8	16.2	− 0.07
Lab							
Serum creatinine, mg/dL	1.478	1.20 ± 0.40	1.07 ± 0.33	0.35	1.16 ± 0.38	1.09 ± 0.34	0.19
eGFR, mL/min/1.73m^2^	1.478	69.3 ± 25.5	77.9 ± 27.7	− 0.32	71.6 ± 26.1	76.4 ± 27.2	− 0.18
NT-Pro BNP, pg/mL	353	1936 [600, 5279]	2141 [749, 4876]	NA	1746 [513, 4562]	2130 [662, 4923]	NA
BNP, pg/mL	709	804 [289, 1551]	697 [289, 1368]	NA	804 [297, 1664]	685 [279, 1320]	NA
HbA1C, %	1.487	7.3 ± 1.4	8.5 ± 1.9	− 0.72	7.6 ± 1.6	8.1 ± 1.9	− 0.30
Sodium (Na), mEq/L	1.196	139.0 ± 4.0	138.7 ± 3.5	0.09	138.9 ± 3.9	138.7 ± 3.6	0.06
Potassium (K), mEq/L	1.327	4.2 ± 0.5	4.1 ± 0.5	0.14	4.2 ± 0.5	4.1 ± 0.5	0.07
Uric acid, mg/dL	1.063	7.0 ± 2.3	6.9 ± 2.2	0.06	6.9 ± 2.2	6.9 ± 2.3	0.04
AST, U/L	944	33.0 ± 21.6	33.2 ± 24.2	− 0.01	32.2 ± 20.7	32.6 ± 23.1	− 0.04
ALT, U/L	1.368	30.2 ± 25.7	31.7 ± 25.5	− 0.06	28.7 ± 24.1	31.5 ± 25.1	− 0.11
LDL-C, mg/dL	1.426	66.8 ± 42.7	66.3 ± 51.8	0.01	68.9 ± 43.4	66.7 ± 50.0	0.05
Total cholesterol, mg/dL	1.376	157.8 ± 39.2	162.2 ± 43.0	− 0.11	159.8 ± 40.9	161.3 ± 42.0	− 0.04
Hemoglobin, g/dL	1.206	13.4 ± 2.2	13.3 ± 2.3	0.06	13.4 ± 2.1	13.3 ± 2.2	0.03
HHF in the previous month	1.487	193 (29.8)	346 (41.2)	− 0.24	32.5	40.2	− 0.16
HHF in the previous year	1.487	353 (54.6)	472 (56.2)	− 0.03	56.2	56.7	− 0.01
Number of HHF in the previous 3 years	1.487						
0		238 (36.8)	329 (39.2)	− 0.05	36.3	38.8	− 0.05
1		258 (39.9)	404 (48.1)	− 0.17	42.8	47.2	− 0.09
≥ 2, frequent		151 (23.3)	107 (12.7)	0.28	20.9	14.0	0.18
Acute pulmonary oedema	1.487	54 (8.3)	65 (7.7)	0.02	7.8	7.6	0.01
Comorbidity							
Hypertension	1.487	488 (75.4)	611 (72.7)	0.06	75.0	72.1	0.07
Coronary artery disease	1.487	356 (55.0)	473 (56.3)	− 0.03	55.1	54.2	0.02
Dyslipidemia	1.487	391 (60.4)	498 (59.3)	0.02	60.2	57.8	0.05
Chronic kidney disease	1.487	257 (39.7)	241 (28.7)	0.23	36.9	31.1	0.12
Myocardial infarction	1.487	186 (28.7)	301 (35.8)	− 0.15	28.6	33.6	− 0.11
Atrial fibrillation	1.487	174 (26.9)	154 (18.3)	0.21	23.5	19.1	0.11
Chronic obstructive pulmonary disease	1.487	154 (23.8)	131 (15.6)	0.21	22.8	15.9	0.18
Stroke	1.487	87 (13.4)	99 (11.8)	0.05	12.7	11.1	0.05
Peripheral artery disease	1.487	73 (11.3)	71 (8.5)	0.10	10.9	8.4	0.09
Liver cirrhosis	1.487	15 (2.3)	20 (2.4)	< 0.01	2.0	2.2	− 0.02
Heart failure agents							
RASi (other than ARNI)	1.487	–	753 (89.6)	–	–	89.7	–
Beta-blocker	1.487	581 (89.8)	756 (90.0)	− 0.01	89.3	89.3	< 0.01
Loop diuretics	1.487	469 (72.5)	526 (62.6)	0.21	71.3	64.8	0.14
MRAs	1.487	371 (57.3)	424 (50.5)	0.14	59.1	52.3	0.14
Nitrates	1.487	286 (44.2)	414 (49.3)	− 0.10	43.1	46.5	− 0.07
DHP-CCB	1.487	103 (15.9)	112 (13.3)	0.07	15.2	12.9	0.06
Alpha-blocker	1.487	72 (11.1)	97 (11.5)	− 0.01	10.5	12.6	− 0.07
Vasodilators	1.487	19 (2.9)	26 (3.1)	− 0.01	2.4	2.9	− 0.03
Thiazides	1.487	16 (2.5)	12 (1.4)	0.08	2.1	1.3	0.07
Hypoglycemic agents							
Metformin	1.487	336 (51.9)	700 (83.3)	− 0.71	62.9	76.6	− 0.30
Sulfonylurea	1.487	188 (29.1)	444 (52.9)	− 0.50	36.4	48.0	− 0.24
DPP4i	1.487	277 (42.8)	317 (37.7)	0.10	45.8	39.6	0.13
Alpha-Glucosidase	1.487	44 (6.8)	103 (12.3)	− 0.19	6.5	11.6	− 0.18
Glinide	1.487	14 (2.2)	35 (4.2)	− 0.11	2.6	4.1	− 0.09
GLP1-RA	1.487	20 (3.1)	13 (1.5)	0.10	3.1	1.4	0.12
Insulin	1.487	80 (12.4)	137 (16.3)	− 0.11	12.8	15.0	− 0.06
Other medications							
Statin	1.487	436 (67.4)	646 (76.9)	− 0.21	69.7	75.4	− 0.13
Aspirin	1.487	340 (52.6)	581 (69.2)	− 0.35	56.6	65.1	− 0.17
P2Y12	1.487	220 (34.0)	391 (46.5)	− 0.26	37.4	43.2	− 0.12
Anticoagulation (NOAC, warfarin)	1.487	155 (24.0)	168 (20.0)	0.10	20.8	20.5	0.01
Digoxin	1.487	102 (15.8)	99 (11.8)	0.12	14.4	12.1	0.07
Amiodarone	1.487	71 (11.0)	60 (7.1)	0.13	10.0	8.1	0.07
Follow up years	1.487	2.5 ± 1.4	2.2 ± 1.5	0.17	2.4 ± 1.4	2.2 ± 1.4	0.15

*ALT* alanine aminotransferase, *ARNI* angiotensin receptor–neprilysin inhibitor, *AST* aspartate aminotransferase, *BNP* B-type natriuretic peptide, *DHP-CCB* dihydropyridine calcium channel blockers, *DPP4i* dipeptidyl peptidase-4 inhibitor, *eGFR* estimated glomerular filtration rate, *GBM* generalized boosted modelling, *GLP1-RA* glucagon-like peptide-1 receptor agonist, *HbA1c* glycohemoglobin, *HHF* hospitalization for heart failure, *IPTW* inverse probability treatment weighting, *LA* left atrium, *LDL-C* low density lipoprotein cholesterol, *LVEDD* left ventricular end-diastolic diameter, *LVEF* left ventricular ejection fraction, *LVESD* left ventricular end-systolic diameter, *MR* mitral regurgitation, *MRAs* mineralocorticoid receptor antagonists, *NOACs* novel oral anticoagulants, *NT-Pro BNP* N-terminal pro B-type natriuretic peptide, *NYHA* New York Heart Association, *P2Y12* purinergic receptor P2Y, G protein–coupled,12, *RASi* renin–angiotensin system inhibitors, *SGLT2i* sodium–glucose cotransporter 2 inhibitors, *STD* standardized difference

^a^Data before GBM-IPTW are presented as frequencies (percentages), means ± standard deviations, or medians [quantile 1, quantile 3]

^b^Data after GBM-IPTW are presented as percentages, means ± standard deviations, or medians [quantile 1, quantile 3]

^c^An absolute standardized difference of < 0.2 indicated a non-substantial difference between groups

## Results

### Patient characteristics and baseline demographics

After applying the exclusion criteria, we identified 1487 patients with concurrent T2DM and HFrEF who underwent ARNI or SGLT2i treatment for the first time between January 2016 and December 2021 (*n* = 647 and 840 in the ARNI and SGLT2i groups, respectively). The baseline demographics and clinical characteristics of the patients are listed in Table 1. Compared with the SGLT2i group, the ARNI group had poorer echocardiography results, poorer renal function, and lower HbA1c levels. Furthermore, they were less likely to have acute heart failure (defined as HHF in the previous month); were more likely to have had ≥ 2 HHFs previously; were more likely to have atrial fibrillation and chronic obstructive pulmonary disease; took more loop diuretics; and took less metformin, sulfonylurea, statins, or antiplatelet agents (absolute STD values of > 0.2). After GBM-IPTW, the balance of the baseline data between the two groups was considerably improved. However, LVESD, HbA1c, metformin use, and sulfonylurea use remained imbalanced as covariates.

### Clinical outcomes

**Fig. 2:**
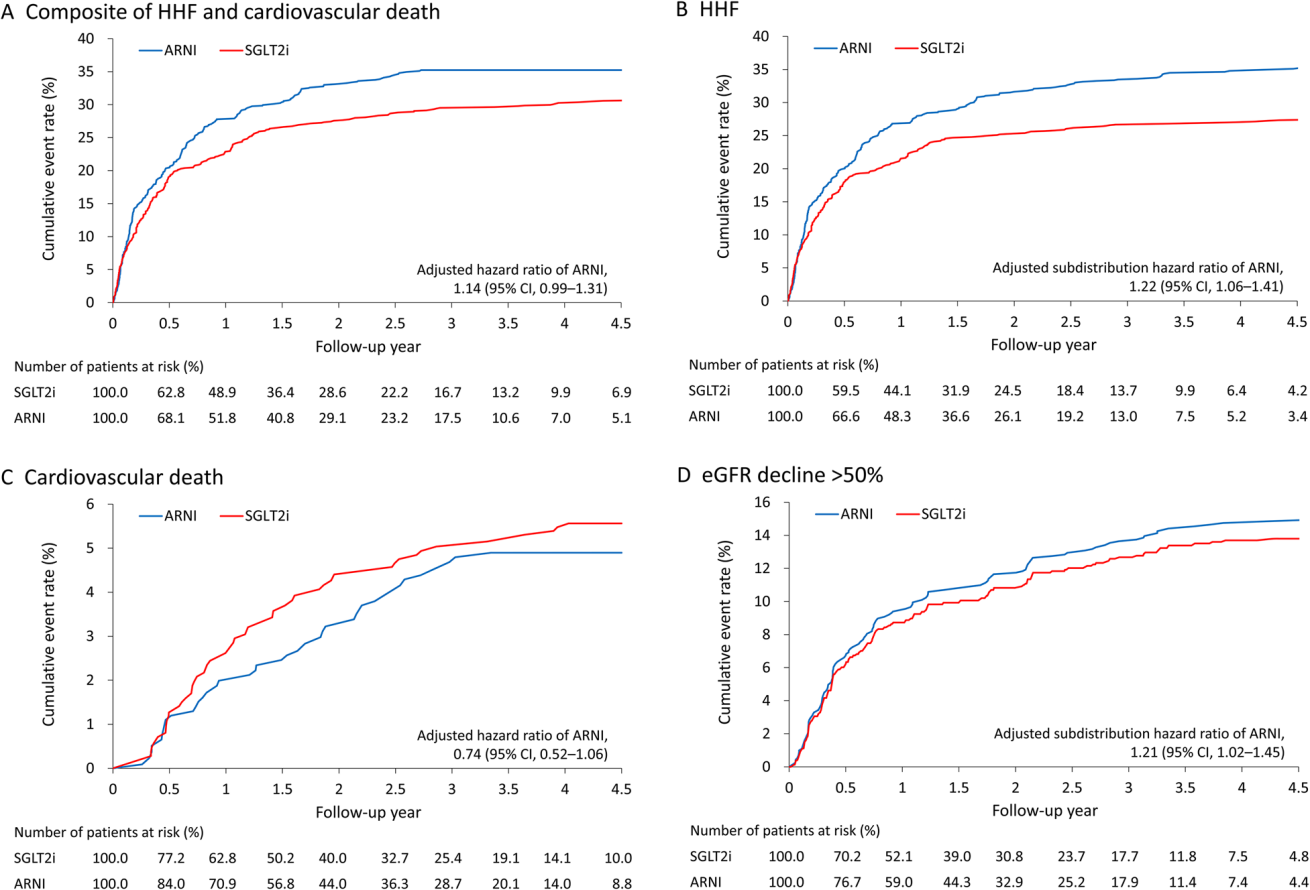
Cumulative event rate of composite outcome of HHF and CV death (**A**), HHF (**B**), CV death (**C**), and an eGFR decline of > 50% during follow-up (**D**) for patients who received ARNI versus SGLT2i treatment in the IPTW-adjusted cohort. *ARNI* angiotensin receptor–neprilysin inhibitor, *CV* cardiovascular, *eGFR* estimated glomerular filtration rate, *HHF* hospitalization for heart failure, *IPTW* inverse probability treatment weighting, *SGLT2i* sodium–glucose cotransporter 2 inhibitors

The mean follow-up was 2.3 years (standard deviation = 1.4 years). During the follow-up, 202 patients (202/647, 31%) in the ARNI group switched to or added SGLT2i treatment, whereas 293 patients (293/840, 35%) in the SGLT2i group switched to or added ARNI treatment (data not shown). The data of the patients who switched to or added on another agent were censored. The clinical outcomes are listed in Table 2. Although a trend toward a higher risk of composite HHF and CV death in the ARNI group was observed (HR 1.14, 95% CI 0.99–1.31,* P* = 0.069), the trend was nonsignificant (Fig. 2A). Notably, the incidence of HHF was significantly greater in the ARNI group than it was in the SGLT2i group (subdistribution HR [SHR] 1.22, 95% CI 1.06–1.41; Fig. 2B). No significant between-group differences in the risks of CV death (Fig. 2C), all-cause mortality, myocardial infarction, or ischemic stroke were noted.

**Table 2:** Follow-up outcomes of patients after inverse probability treatment weighting

Outcome	ARNI	SGLT2i	aHR or aSHR of ARNI (95% CI)^c^	*P*
CV outcome				
Composite of HHF and CV death^a^	258 (39.9)	255 (30.4)	1.14 (0.99–1.31)	0.069
Hospitalization for heart failure^b^	244 (37.7)	228 (27.1)	1.22 (1.06–1.41)	0.006
Cardiovascular death^a^	37 (5.7)	47 (5.6)	0.74 (0.52–1.06)	0.101
All-cause death^a^	88 (13.6)	100 (11.9)	0.98 (0.77–1.24)	0.837
Myocardial infarction^b^	22 (3.4)	24 (2.9)	1.40 (0.88–2.22)	0.159
Ischemic stroke^b^	11 (1.7)	14 (1.7)	0.99 (0.53–1.86)	0.972
Renal outcome				
Serum creatinine doubling^b^	85 (13.1)	78 (9.3)	1.35 (1.05–1.75)	0.022
eGFR decline > 50%^b^	161 (24.9)	168 (20.0)	1.21 (1.02–1.45)	0.034
End-stage renal disease^b^	20 (3.1)	13 (1.5)	2.91 (1.62–5.23)	< 0.001
Potassium (K) > 6 mEq/L^b^	32 (4.9)	21 (2.5)	1.53 (0.99–2.35)	0.055

Data are presented as frequencies (percentages)

*aHR* adjusted hazard ratio, *ARNI* angiotensin receptor–neprilysin inhibitor, *aSHR* adjusted subdistribution hazard ratio, *CI* confidence interval, *CV* cardiovascular, *eGFR* estimated glomerular filtration rate, *HHF* hospitalization for heart failure, *SGLT2i* sodium–glucose cotransporter 2 inhibitors

^a^Cox proportional hazard model with aHR

^b^Fine and Gray subdistribution hazard model with aSHR

^c^Adjusted for baseline LVESD, glycated hemoglobin, use of metformin and sulfonylurea

Compared with the patients who received SGLT2i, the patients who received ARNI had significantly higher risks of all renal outcomes, including doubling of serum creatinine (SHR 1.35, 95% CI 1.05–1.75), an eGFR decline of > 50% (SHR 1.21, 95% CI 1.02–1.45; Fig. 2D), and progression to ESRD (SHR 2.91, 95% CI 1.62–5.23). The risk of hyperkalemia (> 6 mEq/L) during follow-up was borderline significantly greater in the ARNI group (SHR 1.53, 95% CI 0.99–2.35, *P* = 0.055).

**Fig. 3:**
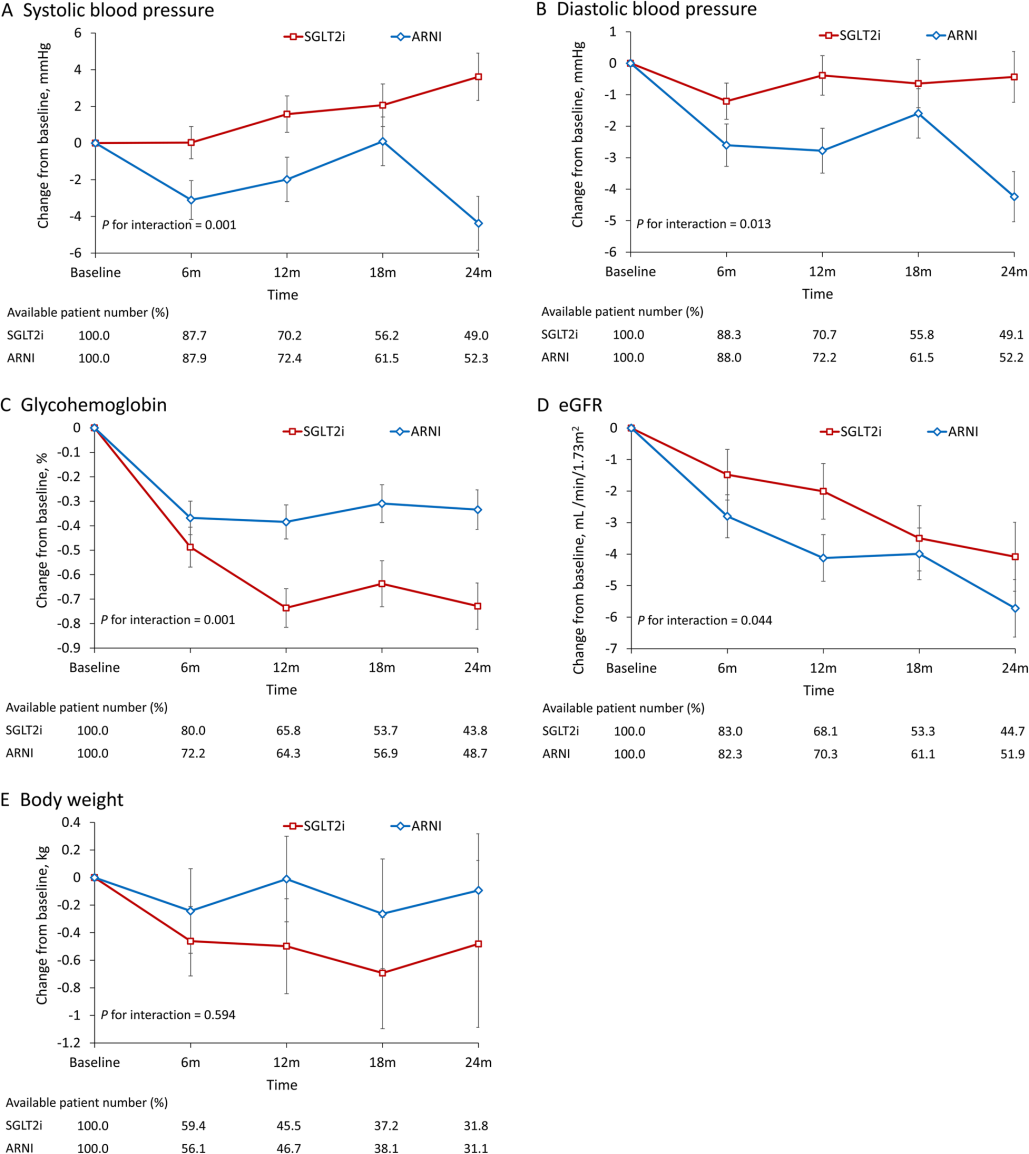
Mean and standard error of systolic blood pressure (**A**), diastolic blood pressure (**B**), glycated hemoglobin (**C**), eGFR (**D**), and body weight (**E**) of follow-up measurements of patients undergoing ARNI versus SGLT2i treatment in the IPTW-adjusted cohort. *ARNI* angiotensin receptor–neprilysin inhibitor, *IPTW* inverse probability treatment weighting, *SGLT2i* sodium–glucose cotransporter 2 inhibitors

**Fig. 4:**
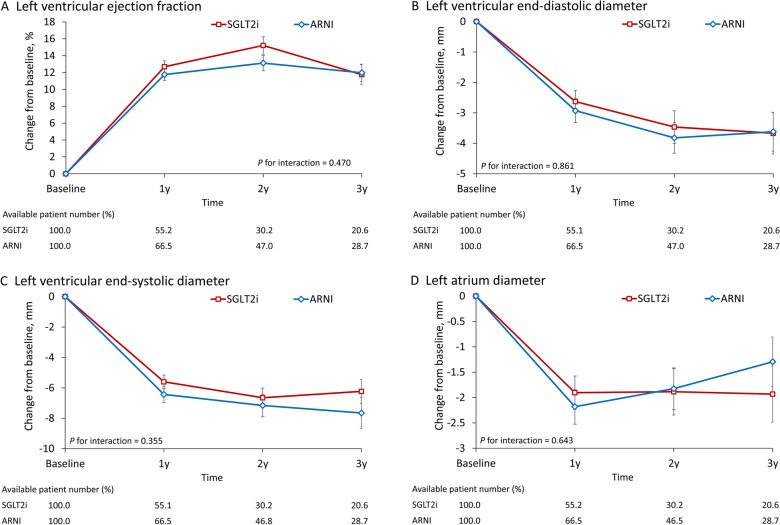
Mean and standard error of left ventricular ejection fraction (**A**), left ventricular end-diastolic diameter (**B**), left ventricular end-systolic diameter (**C**), and left atrium diameter (**D**) during follow-up measurements of patients who received ARNI versus SGLT2i treatment in the IPTW-adjusted cohort. *ARNI* angiotensin receptor–neprilysin inhibitor, *IPTW* inverse probability treatment weighting, *SGLT2i* sodium–glucose cotransporter 2 inhibitors

We also analyzed changes in blood pressure, HbA1c, eGFR, body weight, LVEF, LVEDD, LVESD, and LA diameter. The ARNI group had a greater reduction in blood pressure, including systolic blood pressure (*P* for interaction = 0.001; Fig. 3A) and diastolic blood pressure (*P* for interaction = 0.013; Fig. 3B), from baseline to follow-up. Moreover, the SGLT2i group exhibited a greater reduction in HbA1c (*P* for interaction = 0.001; Fig. 3C) at follow-up. In both groups, the eGFR declined with time during the follow-up period; however, the SGLT2i group retained its renal function to a significantly greater extent than did the ARNI group (*P* for interaction = 0.044; Fig. 3D). The changes in body weight were comparable between groups (Fig. 3E). In both groups, the LVEF improved relative to the baseline to a comparable extent (Fig. 4A). No significant difference in LVEF improvement between the two treatments was noted (P = 0.470). Variations in left ventricular (LV) diameter and left atrium (LA) were analyzed during the follow-up period. The LVESD, LVEDD, and LA diameters all decreased over the treatment period, with no notable between-group differences (P = 0.861, 0.355, and 0.643 for LVEDD, LVESD, and LA, respectively; Fig. 4B–D).

## Discussion

This is the first study to compare the clinical outcomes of ARNI and SGLT2i treatments among patients with concurrent HFrEF and T2DM. The study indicated that SGLT2i treatment conferred significant benefits in reducing HHF and providing greater renal protection compared to ARNI treatment but comparable effects on CV death, myocardial infarction, ischemic stroke, and heart remodeling. These findings may serve as a reference for optimizing guideline-directed medical therapy (GDMT) in patients with concurrent HFrEF and T2DM.

Our results demonstrated that SGLT2i provided greater protection against HHF than ARNI. Clinical comparisons of these two categories in patients have yet to be completed in major trials. Aimo et al. performed a network meta-analysis to compare the outcomes of ARNI, vericiguat, and SGLT2i treatments in patients with HFrEF in major trials. They reported that these treatments resulted in comparable reductions in the risk of CV death or HHF [16]. However, a 2022 systematic review and network meta-analysis revealed the superiority of ARNI to SGLT2i in the composite of HHF and CV death and more significant blood pressure reduction in patients with HFrEF [17]. The mechanisms of HHF difference possibly result from the pharmacophysiology of these two drugs. ARNI was developed to synergistically enhance the natriuretic peptide system while blocking the renin–angiotensin–aldosterone system (RAAS). By promoting cardiac remodeling and reducing fibrosis, ARNI relies on the actions of various endopeptides, such as natriuretic peptides, adrenomedullin, substance P, and the products of angiotensin I and II cleavage by neprilysin. On the other hand, SGLT2i medications exhibit numerous pharmacodynamic properties that could impact cardiovascular outcomes in patients with heart failure. These effects involve glycosuria, osmotic diuresis, and natriuresis, reductions in arterial stiffness, blood pressure, and pulmonary vascular resistance, elevated hemoglobin levels, anti-inflammatory and anti-oxidant actions, and cardioprotective and renoprotective effects [18]. SGLT2i may reduce HHF more effectively than ARNI due to its unique mechanism of action. SGLT2i influences volume redistribution in the proximal tubules, which could have a greater impact on reducing cardiac workload compared to the synergistic inhibition of natriuretic peptides and the RAAS provided by ARNI. This difference in mechanism may contribute to the varying outcomes observed between these two treatment options. ARNI or SGLT2i can be employed as an add-on treatment for GDMT with beta-blockers and ACEI/angiotensin receptor blockers [4, 8–11, 16–18]. In clinical practice, numerous patients are unable to utilize both drugs simultaneously due to hypotension or deconditioning. Additionally, in many regions, the financial burden or insurance limitations associated with the concomitant use of both drugs can be prohibitive, necessitating selecting one of the two medications. Consequently, determining which drug to prioritize in patients with HFrEF coexisting with diabetes has emerged as a pressing, practical issue that must be addressed. Our real-world analysis observed that SGLT2i is superior in HHF than SGLT2i but has no differences in CV death, all-cause mortality, myocardial infarction, or ischemic stroke. When T2DM combined HFrEF patients and physicians encounter the dilemma of optimized GDMT and cost, prioritizing SGLT2i could prove advantageous in HHF and renal outcomes.

Renal function deterioration is a serious concern for patients with HFrEF. We found that SGLT2i conferred more favorable renal protection than ARNI in doubling serum creatinine, an eGFR decline of > 50%, and progression to ESRD. In PARADIGM-HF and a meta-analysis by Kang et al., ARNI use was associated with improved renal outcomes [4, 19, 20]. Yip et al. also demonstrated the protective role of ARNI against cardiorenal syndrome–induced kidney damage in an animal study [21]. The possible mechanism of ARNI-preserved eGFR included decreasing renal perfusion, increasing natriuretic peptide, or just physiologic response in heart failure status [22]. SGLT2i treatment has also shown renoprotective effects, observed in main trials on T2DM [5–7]. The renoprotective benefits of SGLT2i include reduced glomerular hyperfiltration, microvascular and macrovascular protection, cardiac benefits, weight reduction, and reduced sympathetic activity [23, 24]. The EMPEROR-Reduced trial and real-world database analysis revealed that empagliflozin significantly reduced the risk of renal disease progression [9, 25]. The DAPA-HF trial enrolled HFrEF patients, with 40.6% having CKD [8]. Subgroup analysis from the DAPA-HF trial indicated that dapagliflozin exerted significantly greater renoprotective effects than the placebo, characterized by a slowed eGFR decline [8, 26]. There have been no real-world comparisons of the renoprotective effects of ANRI and SGLT2i treatments in patients with concurrent HFrEF and T2DM. Although ARNI and SGLT2i have exhibited renal benefits in previous studies, our findings indicated that SGLT2i treatment might provide superior renal protection for patients with coexisting HFrEF and T2DM. This could be attributed to the distinctive mechanisms of action of SGLT2i, such as decreased glomerular hyperfiltration, improved microvascular and macrovascular protection, and earlier direct effects in the glomerular. Further research is needed to confirm these findings and elucidate the reasons for this difference.

In addition to clinical cardiovascular events, we evaluated heart remodeling in patients following ARNI versus SGLT2i treatment, a process that may benefit clinical prognosis, including reduced mortality and rehospitalization risks. Our finding showed improvements in LVEF and reduction in LV and LA size in both groups, with no significant differences between them. However, previous studies on cardiac remodeling effects for both medications have shown inconsistent results. One study reported significant improvement in LVEF and heart remodeling after over a year of ARNI treatment, with LVEF increasing from 28.2 to 37.8%. This was accompanied by substantial reductions in left ventricular end-systolic and left ventricular end-diastolic volume indexes [27]. In contrast, evidence on the effects of SGLT2i is mixed. The REFORM trial involved 56 patients with concurrent diabetes mellitus and HFrEF found no significant change in LVEF or cardiac size after a year of dapagliflozin treatment [28]. Another randomized study by Omar et al., including 190 patients with HFrEF, reported significant reductions in left ventricular end-systolic, left ventricular end-diastolic, and LA volume indexes after 12 weeks of empagliflozin treatment but no change in LVEF [29]. The mechanisms behind cardiac remodeling by SGLT2i could involve several factors, such as improved ventricular loading due to reduced preload and afterload, enhanced cardiac metabolism and bioenergetics, inhibition of myocardial Na^+^/H^+^ exchange, reduction of necrosis and fibrosis, and alterations in adipokine and cytokine production, as well as epicardial adipose tissue mass [30]. Despite the different mechanisms of cardiac remodeling for ARNI and SGLT2i, our follow-up data indicated comparable LVEF and cardiac remodeling parameters between the two treatment groups. Compared to previous SGLT2i studies, our study had more extended follow-up periods, lower baseline LVEF, and higher GDMT achieved rates, including 89.6% with RASi. These factors could contribute to the more favorable cardiac remodeling effects observed in our SGLT2i group, ultimately resulting in comparable cardiac remodeling outcomes with the ARNI treatment. However, further investigation into the cardiac remodeling effect of SGLT2i is warranted.

We assessed blood pressure, HbA1c, and body weight changes over the follow-up period. The ARNI group experienced a greater reduction in systolic and diastolic blood pressure than the SGLT2i group. While SGLT2i has a diuretic effect that can lower blood pressure, ARNI combines the neprilysin inhibitor sacubitril with the angiotensin receptor blocker valsartan, which is well-known for its hypotensive effect. Additionally, despite the SGLT2i group having a higher baseline HbA1c level, it showed a more substantial reduction during treatment. This improvement may be attributed to the treatment itself or possibly due to physicians in the SGLT2i group adopting a more aggressive approach to glucose control, given the higher baseline HbA1c. Previous studies have reported reductions in body weight for patients undergoing SGLT2i treatment [31, 32]. In our study, the SGLT2i group demonstrated a greater decrease in body weight than the ARNI group during the follow-up period, although the difference was not statistically significant (*P* = 0.725).

Our study has several limitations that should be considered when interpreting the results. First, the real-world evidence and the study’s retrospective nature precluded random selection; selection bias and inherent differences are potential concerns. Because of this, we adjusted for most of the covariates that might be related to the outcomes with GBM-IPTW matching methods. However, some differences existed, including baseline HbA1C and the use of hypoglycemic agents. Compared with the ARNI group, the SGLT2i group had a higher average baseline HbA1c level (7.6 vs. 8.1; STD = − 0.30), and the hypoglycemic agents used by the two groups differed. In addition, a greater proportion of patients in the SGLT2i group took metformin and sulfonylurea, which are considered to have neutral effects on HFrEF. Second, the causal relationship in clinical practice is also difficult to verify in observational studies. Nevertheless, we enrolled patients who received SGLT2i and ARNI and evaluated the same parameters and outcomes in both groups. Therefore, the causal relationship should be relatively valid in this study. Third, although we analyzed the parameters of heart remodeling, there are still numerous deficiencies and a need for further calibration within the database in assessing and measuring heart function, including diastolic function. Furthermore, the database contained no information on physical activity, personal habits, and functional statuses, all of which can affect the prognosis of patients with HFrEF and T2DM. Finally, medication noncompliance may have occurred, and the data obtained on the patients’ prescriptions may not have reflected the patients’ actual medication use.

## Conclusion

SGLT2i treatment was associated with more significant HHF risk reduction and protection against renal function decline than ARNI treatment. In situations where patients' medical conditions or financial resources must be considered, prioritizing the use of SGLT2i may be beneficial. These findings reinforce existing treatment guidelines and could assist healthcare professionals in selecting the most appropriate medication for patients with concurrent HFrEF and T2DM.

## Supplementary Information

**Additional file 1: Fig S1A. Level of BNP changes. **ARNI, angiotensin receptor–neprilysin inhibitor; BNP, B-type natriuretic peptide; SGLT2i, sodium–glucose cotransporter 2 inhibitors.

**Additional file 2: Fig S1B. Level of NT-Pro BNP changes. **ARNI, angiotensin receptor–neprilysin inhibitor; NT-Pro BNP, N-terminal pro B-type natriuretic peptide; SGLT2i, sodium–glucose cotransporter 2 inhibitors.

**Additional file 3.** Supplementary material.

## Data Availability

The original contributions presented in this study are included in the article and Additional file 3. Further inquiries can be directed to the corresponding author.
