# Supplementary figures and images for: Comparing angiotensin receptor–neprilysin inhibitors with sodium–glucose cotransporter 2 inhibitors for heart failure with diabetes mellitus
Source: Diabetol Metab Syndr. 2023 May 26;15:110. doi: 10.1186/s13098-023-01081-2 (PMC10214563; doi:10.1186/s13098-023-01081-2)

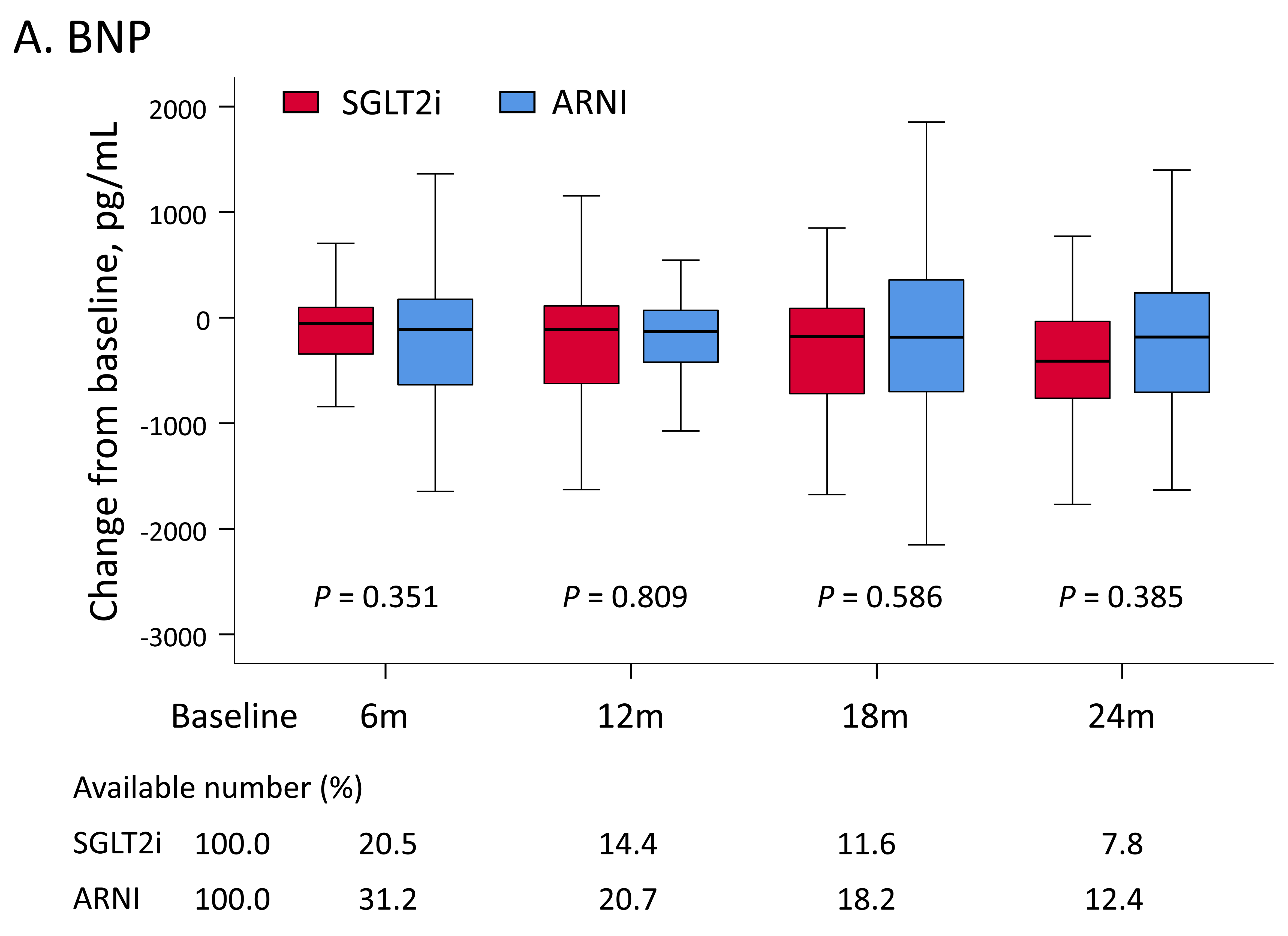

Supplement: Supplementary file 1 — Additional file 1: Fig S1A. Level of BNP changes. ARNI, angiotensin receptor–neprilysin inhibitor; BNP, B-type natriuretic peptide; SGLT2i, sodium–glucose cotransporter 2 inhibitors. [file 13098_2023_1081_MOESM1_ESM.tif]

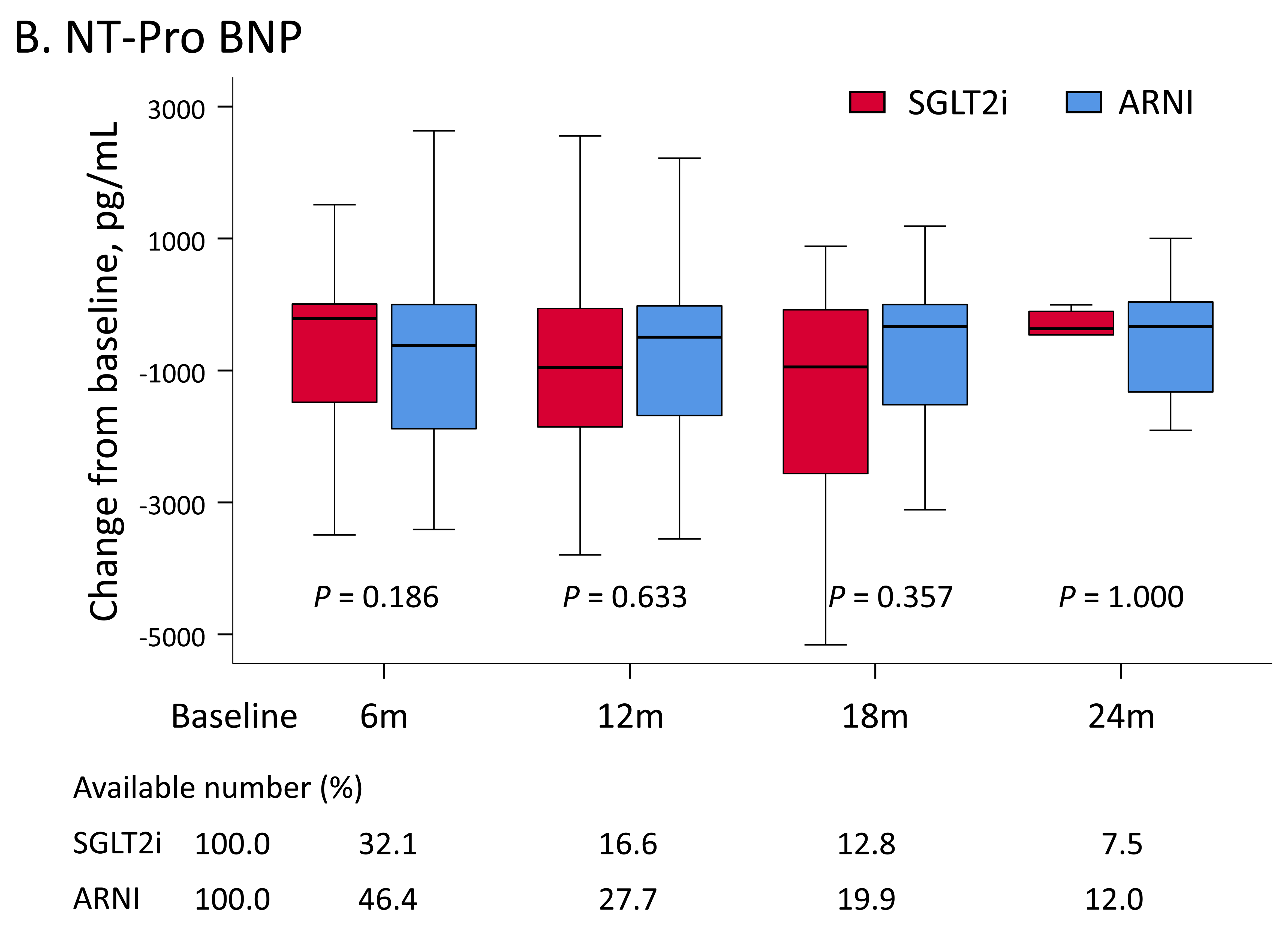

Supplement: Supplementary file 2 — Additional file 2: Fig S1B. Level of NT-Pro BNP changes. ARNI, angiotensin receptor–neprilysin inhibitor; NT-Pro BNP, N-terminal pro B-type natriuretic peptide; SGLT2i, sodium–glucose cotransporter 2 inhibitors. [file 13098_2023_1081_MOESM2_ESM.tif]
