# Supplementary material for: Comparing angiotensin receptor–neprilysin inhibitors with sodium–glucose cotransporter 2 inhibitors for heart failure with diabetes mellitus
Source: Diabetol Metab Syndr. 2023 May 26;15:110. doi: 10.1186/s13098-023-01081-2 (PMC10214563; doi:10.1186/s13098-023-01081-2)
Supplement: Supplementary file 3 — Additional file 3. Supplementary material. [file 13098_2023_1081_MOESM3_ESM.docx]

**Supplementary material**

**Covariate measurements**

The covariates of interest were demographic characteristics (age, sex, smoking status, and body mass index), baseline vital signs (systolic and diastolic blood pressure and heart rate), HHFs in the previous month (as a proxy for acute failure) and HHFs in the previous year, the number of HHFs in the previous 3 years, comorbidities, medications used during the treatment period, laboratory test results (including HbA1c and serum creatinine levels), and echocardiography results. The echocardiographic parameters of interest were the LVEF, left ventricular end-diastolic diameter (LVEDD), left ventricular end-systolic diameter (LVESD), left atrial diameter (LA), and mitral regurgitation severity. Data at baseline (6 months prior to the index date) on body mass index, vital signs, laboratory results, and medications were collected.

**Outcome definitions**

Outcomes comprised clinical events and continuous outcomes (e.g., blood pressure and echocardiographic parameters). Clinical events comprised CV and renal outcomes. CV outcomes were a composite of CV death or HHF, HHF, CV death, all-cause mortality, myocardial infarction, and ischemic stroke. Renal outcomes comprised doubling of serum creatinine, an eGFR decline of >50%, end-stage renal disease (ESRD), and hyperkalemia (potassium of >6 mEq/L) during follow-up. HHF was defined as worsening heart failure requiring hospitalization in which the patient required at least one treatment, which may have included diuretics, nitrites, or inotropic agents. Causes of death were linked to the information in the Taiwan Death Registry database. CV death was defined as death due to acute myocardial infarction; sudden cardiac death; or death due to heart failure, stroke, CV procedures, CV hemorrhage, or other CV causes. Patients were followed from the index date to the date of death; outcome occurrence; the addition of or a switch to the other agent (e.g., ARNI to SGLT2i or ARNI added onto SGLT2i treatment); the date of the most recent visit in the CGMH system; or December 31, 2021, whichever occurred first.

Data on systolic blood pressure, diastolic blood pressure, HbA1c, eGFR, and body weight were extracted at 6-, 12-, 18-, and 24-month follow-ups. In addition, echocardiographic parameters (LVEF, LVEDD, LVESD, and LA) were extracted at 1-, 2-, and 3-year follow-ups.

**Statistical analysis**

The risk of fatal time-to-event outcomes (e.g., CV death) between the two groups was compared using Cox proportional hazard modelling. The incidence of nonfatal time-to-event outcomes (e.g., HHF, an eGFR decline of >50% during follow-up) between groups was compared using the Fine–Gray subdistribution hazard model, with all-cause mortality during follow-up considered a competing risk. The between-group changes between the baseline and follow-up measurements of the continuous outcomes (e.g., LVEF) were compared using a linear mixed model with random intercept. Time was regarded as a continuous variable, and the interaction between time and the two groups (ARNI vs. SGLT2i) was considered in the mixed model. Due to the potential imbalance in covariates (LVESD, HbA1c, and use of metformin and sulfonylurea; absolute STD values of >0.2) between groups even after GBM-IPTW, adjustments were made in the regression models.

**Supplementary Figure 1A. Level of BNP changes.**

ARNI, angiotensin receptor–neprilysin inhibitor; BNP, B-type natriuretic peptide; SGLT2i, sodium–glucose cotransporter 2 inhibitors.

**Supplementary Figure 1B. Level of NT-Pro BNP changes.**

ARNI, angiotensin receptor–neprilysin inhibitor; NT-Pro BNP, N-terminal pro B-type natriuretic peptide; SGLT2i, sodium–glucose cotransporter 2 inhibitors.
